# The Reaction of EDTA With Metallothionein and its Separate Clusters: Complex Kinetic Behavior With Novel Protein Concentration Dependence

**DOI:** 10.1155/MBD.1994.524

**Published:** 1994

**Authors:** Amalia Munoz, David H. Petering, Gan Tong, C. Frank Shaw III

**Affiliations:** 1 Department of Chemistry University of Wisconsin-Milwaukee Milwaukee WI 53201-0413 USA


					THE REACTION OF EDTA WITH METALLOTHIONEIN
AND ITS SEPARATE CLUSTERS: COMPLEX KINETIC BEHAVIOR
WITH NOVEL PROTEIN CONCENTRATION DEPENDENCE
Amalia Munoz, David H. Petering, Gan Tong and C. Frank Shaw III
Department of Chemistry, University of Wisconsin-Milwaukee, Milwaukee, WI-53201-0413, USA
Mammalian metallothionein (MT) is Involved in the detoxiflcation of Cd(ll) ions. It binds 7 Cd(ll) in
two Cd-thiolate clusters (Cd4S11 and C3S9) which are located in two protein domains. 111Cd and
2-D NMR studies show that in lobster, two Cd3S9 domains exist. Despite homologies in the
location of cysteines between the corresponding domains of the lobster and mammalian MTs (8 of
the 9 cysteines in each domain), the sequence specific Cd-thiolate connections differ in both
domains of the lobster protein. To see if these differences alter the chemistry of the proteins, two
reactions related to the function of the protein were compared.
Mammalian Cd7-MT reacts with EDTA with biphasic kinetics to form Cd-EDTA and apoMT. The
dedvcl rate law is consistent with the initial ormtion ot a C67-MT-EDTA adduct.
k'[EDTA]
kobs k +
KD + [EDTA]
Examination of the reactions of isolated x- and 8-domains with EDTA show generally similar kinetics,
but demonstrate that for the e-domain that cadmium removal proceeds through a partially Cd-
depleted intermediate Cd3S11" According to 111Cd NMR titration ofthe holo-protein, shows a slight
preference for removing Cd from the 3-metal cluster.
The metal exchange between apoMT with Cd-substituted carbonic anhydrase was considerably
faster than that Involving EDTA, with 30-50% exchange occurring in 30 min. Comparative reactions
with lobster MT will also be described. (Supported by NIH grants ES-04026 and ES-4184).
524

				

